# Examining variation in the relationship between disability and physical activity across Australian local government areas

**DOI:** 10.1057/s41271-024-00487-0

**Published:** 2024-05-30

**Authors:** Jerome N. Rachele, George Disney, Allison Milner, Rees Thomas, Jacqueline Le Busque, Rebecca A. Reid, Anne M. Kavanagh

**Affiliations:** 1 College of Sport, Health and Engineering, Victoria University, Footscray, Australia; 2 Institute for Health and Sport, Victoria University, Footscray, Australia; 3 Melbourne School of Population and Global Health, The University of Melbourne, Melbourne, Australia

**Keywords:** Disability studies, Exercise, Epidemiology, Multilevel analysis, Physical activity

## Abstract

Understanding the relationship between disability and physical activity and whether it differs across local government jurisdictions may aid in the development of placed-based approaches to reducing disability-related inequalities in physical activity. The objectives of this study were to examine the association between disability and physical activity and assess whether this association varied between Australian Local Government Areas. The sample included 13,315 participants aged 18–64 years from the Household Income and Labour Dynamics Australia Survey, 2017. Participants self-reported disability and physical activity. Linear mixed-effects models estimated the association between disability and physical activity. People with disability reported less physical activity per week. We did not find evidence that this association varied across LGAs. Our findings do not add evidence towards local government-based approaches in Australia to reducing physical activity inequalities between people with and without a disability.

## Key messages

- Understanding the relationship between disability and physical activity may aid in the development of place-based approaches to reducing disability-related inequalities in physical activity.
- Our study improves our understanding of the role of local government in reducing physical activity disparities between individuals with and without disabilities.

## Introduction

It is well established that regular physical activity is good for health [1]. There is a need for population-level physical activity data on people with disabilities [2]. According to the 2017–2018 Australian National Health Survey, of the participants who reported a disability (24% of the surveyed sample), 27% reported not engaging in any physical activity compared to 10% of people without a disability [3]. This difference is likely to lead to inequalities in health outcomes between those with and without a disability.

Evidence suggests that the extent of health inequalities are likely to differ depending on the environments in which people live [4]. Epidemiological studies that report the average difference in health behaviours for people with different individual characteristics implicitly assume that these differences are the same in all areas. However, it is possible the associations under investigation, and the drivers of these associations, vary between areas. For associations between disability and physical activity, these may include those related to the built and natural environment, economic issues, equipment barriers, and facility and community-level policies and services [5]. Such barriers to physical activity may also vary depending on the type of disability. For example: for people with physical and mobility disability, barriers may include those related to street surfaces, kerb design, and footpath width; for people with sensory disability, navigation around recreational facilities; and for those with intellectual disability, the method of communication (Easy English, a simple everyday language with minimal grammar) on facilities and event websites [6].

Understanding whether inequalities in physical activity between those with and without a disability vary across geographies is important for devising strategies to reduce these inequalities. One such geography of note in Australia is the local government area (LGA). In Australia, local governments are responsible for the maintenance of local roads and footpaths, and facilitate access to healthcare, public transport, sport and recreation, and community services [7].

Identifying whether the association between disability and physical activity varies across local governments is a necessary first step towards further investigation into the potential drivers of any variation, especially local government policy interventions to reduce inequalities. This study aimed to examine the average association between disability and physical activity and assess whether the average association observed between disability and physical activity varied between Australian LGAs.

## Methods

### Data sources

This cross-sectional analysis used data from Wave 17 (2017) of the Household, Income and Labour Dynamics in Australia (HILDA) Survey [8]. The sample included 13,792 respondents from the main sample (including 63.7% from the initial wave) and 3,779 from the top-up sample, totalling 17,571 participants residing in private dwellings in Australia, and the sample is considered representative of the Australian population [9]. We selected sample households at baseline using a multi-staged approach, whereby we selected census collection districts, and from within each area, we selected 22 to 34 dwellings, with up to three households per dwelling selected to be a part of the sample [10]. We collected data between late 2016 and the end of February 2017, with most interviews conducted in the two months of August and September (late winter and early spring).

The University of Melbourne Human Research Ethics Committee approved the HILDA Survey. Informed consent to participate in the HILDA Survey was obtained using an information letter to all potential respondents. This letter described the voluntary nature of participation in the HILDA Survey and outlined that informed consent would be implied when participants agreed to be interviewed.

### Outcome variables: physical activity

We assessed physical activity using the International Physical Activity Questionnaire (IPAQ) Short Form. We asked participants about three types of physical activity: walking, moderate-intensity activity, and vigorous-intensity activity. We edited the data collected and processed it according to rules recommended in the Guidelines for Data Processing and Analysis of the International Physical Activity Questionnaire (IPAQ) short form [12]. We measured total activity time for each activity type in metabolic equivalent of task (or MET) minutes, then summed them to create a METmins variable we used for analysis. Under this measure of physical activity, 10 min of walking would be 33 METmins, 10 min of moderate intensity physical activity 40 METmins, and 10 min of vigorous-intensity physical activity 80 METmins. The physical activity measure we used in this study is similar to those that other researchers have examined among people with disabilities and shown to have comparable test–retest reliability and criterion validity to those used in the general population [13].

### Exposure variable: disability

Disability in HILDA is self-reported and defined as a long-term health condition, impairment or disability that restricts you in your everyday activities and has lasted or is likely to last for 6 months or more. This definition is derived from the International Classification of Functioning (ICF) Disability and Health Framework [11]. We offered specific examples of long-term conditions including limited use of fingers or arms, or problems with eyesight that could not be corrected with glasses or contact lenses.

### Analysis

To reduce the risk of reverse causation: that is, physical inactivity over the life-course causing disability in later life, we restricted the sample to those aged between 18 and 64 years. Following omission of those out-of-scope due to age, 14,305 participants remained. We omitted further participants for missing data on disability status (*n* = 794), physical activity (*n* = 154), income (*n* = 35) and those for whom we were unable to determine their Local Government Area (*n* = 7) leaving a sample of 13,350, 93.3% of in-scope participants.

### Area-level measures

We compiled a four-level geographical data structure as follows:

- Level 1. Study Participants: 13,315 participants.
- Level 2. Sample Households: 7,721 households with a median of 2 participants (interquartile range 1, 6), ranging from 1 to 7.
- Level 3. Local Government Areas: Each LGA is located within one State and Territory only. The functions of local government may include: community facilities such as libraries and parks, maintenance of local roads and footpaths, building regulation and development, local environmental issues, and waste disposal [14]. There were 568 LGAs defined in the 2011 Census, and 438 included in this sample. There was a median (interquartile range) of 15 (1, 168) participants (ranging from 1 to 603) per LGA.
- Level 4. States and Territories: Australia contains eight main States and Territories as separate spatial units. Major responsibilities include schools, hospitals, conservation and environment, roads, railways and public transport, public works, agriculture and fishing, industrial relations, community services, sport and recreation, consumer affairs, police, prisons and emergency services [14]. There was a median (interquartile range) of 1,165 (1126, 1204) participants (ranging from 115 to 3,828) per State and Territory.

We performed separate mixed-effects variance components models with physical activity and disability as the outcomes to examine between-LGA differences in physical activity, and the likelihood of disability. We conducted likelihood ratio tests to compare three-level variance components models specifying States and Territories at level 3, households at level 2, and individuals at level 1, with four-level variance components models specifying States and Territories at level 4, LGAs at level 3, households at level 2, and individuals at level 1. A significant likelihood ratio test denoted between-LGA differences in each of physical activity, and the likelihood of disability. We then performed linear mixed-effects modelling to estimate the association between disability and physical activity. We did this in two stages. First, we fit four-level random intercept model to determine the average difference in physical activity by disability status, specifying States and Territories at level 4, LGAs at level 3, households at level 2, and individuals at level 1. Second, we specified a random coefficient for disability to determine whether the differences in physical activity by disability status varied between LGAs, as determined by a likelihood ratio test.

Postulated relationships between covariates and disability and physical activity informed selection of confounders. We adjusted all models for age, sex, educational attainment, employment status, and income. We performed all analyses using the statistical package Stata version 16 [15].

## Results

We present descriptive statistics in Table 1. Participants with a disability comprised 24.7% of the sample. The mean (standard deviation) physical activity was 2130 (2853) METmins among those with a disability, and 2663 (2916) among those without a disability. The variance components models incorporating LGAs were better fits than their counterpart models without LGAs, denoting significant between-LGA differences for both physical activity (LR *χ*^2^ (1) = 204.31, *p* < 0.001) and disability (LR *χ*^2^ (1) = 130.51, *p* < 0.001).

**Table 1:** Sociodemographic characteristics among participants with and without a disability in the HILDA Study (Wave 17, 2017)

	Disabled	Not disabled
Total participants	3285	10,030
Physical activity^a^ (mean (SD))	2130 (2853)	2663 (2916)
Equivalised household income^b^ (mean (SD))	48,539 (26,619)	60,305 (35,639)
Age, years (mean(SD))	44.3 (13.8)	38.5 (13.1)
*Sex %*		
Male	44.7	48.4
Female	55.4	51.6
*Education %*		
Bachelor degree or higher	20.8	32.6
Advanced diploma, diploma, certificate or year 12	52.2	53.0
Year 11 or below	27.1	14.4
*Employment %*		
Permanent full time	31.3	50.4
Casual (full time or part time)	11.3	14.3
Fixed Term	4.4	7.3
Self-employed / not easily classifiable	53.1	28.1
*Household structure %*		
Couple no children	26.3	25.2
Couple with children	39.0	48.6
Lone parent with children	12.4	8.4
Lone person	16.5	12.8
Other	5.9	4.9

^a^Measured in METmins, ^b^measured in Australian Dollars

Participants with a disability had a mean physical activity of 2130 METmins, with a standard deviation of 2853, variance of 8,138,713, skewness of 2.3 and kurtosis of 9.2. Participants without a disability has a mean physical activity of 2663, with a standard deviation of with a 2916, variance of 8,510,224, skewness of 2.0 and kurtosis of 7.7. People with a disability had an estimated mean difference of − 413 METmins per week (95% confidence interval − 529, − 298) compared to those without a disability: around 13 min of walking intensity physical activity per week. The random effects of the models did not provide evidence that this relationship varied across LGAs (LR *χ*^2^ (2) = 1.57, *p* = 0.456). Fixed and random effects for each of the models are available in Tables 2 and 3, respectively.

**Table 2:** Fixed effects estimates (95% CI) derived from random intercept model of disability and physical activity*: the Household Income and Labour Dynamics in Australia (HILDA) Survey, 2017

Not disabled	Ref
Disabled	− 417 (− 532, − 301)
Equivalised household income^a^	14 (− 1, 30)
Age (years)	− 19 (− 22, − 15)
*Sex*	
Male	Ref
Female	− 1187 (− 1278, − 1096)
*Education*	
Bachelor degree or higher	Ref
Advanced diploma, diploma, certificate or year 12	570 (455, 685)
Year 11 or below	601 (446, 756)
*Employment*	
Permanent full time	Ref
Casual (full time or part time)	322 (175, 469)
Fixed Term	101 (− 94.9, 296)
Self-employed / not easily classifiable	− 256.77 (− 369, − 145)
*Household structure*	
Couple no children	Ref
Couple with children	− 28 (− 208, 44)
Lone parent with children	− 131 (− 323, 61)
Lone person	193 (29, 357)
Other	4 (− 249, 257)

**Table 3:** Random effects estimates (95% CI) derived from multilevel models of disability and physical activity*: the Household Income and Labour Dynamics in Australia (HILDA) Survey, 2017

	Model 1	Model 2
State	247 (119, 515)	247 (119, 514)
Local government area	633 (547, 734)	662 (564, 776)
Disability variance	–	196 (29, 1322)
Covariance	–	− 0.52 (− 0.93, 0.47)
Household	957 (862, 1063)	955 (860, 1062)
Individual	2556 (2512, 2601)	2555 (2511, 2601)
Log likelihood	− 214,355	− 124,354

*Models adjusted for age, sex, education, employment and equivalised household income

## Discussion

This study examined the association between disability and physical activity, and whether this association varied across Australian LGAs. Our finding that adults with a disability had somewhat lower levels of physical activity is consistent with other data from Australia [3], The United States [16], Canada [17], and Norway [18]. Levels of physical activity in this sample were comparable to the Australian population [19]. The reasons for between-country consistency in the association between disability and physical activity are also likely to be similar: personal and environmental barriers associated with disability restricting access to physical activity venues and services [20]. Personal barriers may include pain, fatigue, self-consciousness about exercising in public, the perception that exercise is too difficult, financial limitations, lack of awareness about options, and emotional and psychological barriers [5, 21, 22]. Environmental barriers are likely to include lack of transportation, lack of accessible parks and trails, lack of accessible exercise equipment, unqualified staff who cannot modify or adapt individual and group exercise classes for people with disability, programme and equipment costs, and discriminatory practices at fitness centres and other recreational venues [5].

Strengths of this investigation are use of a large nationally-representative sample, including the large number of clusters and people with a disability. Limitations include use of self-report assessments susceptible to response bias (such as social desirability effects). The study may also be affected by dependent measurement error because both disability and physical activity are self-reported and errors in measurement are likely to be correlated due to individual-level factors such as personality type (agreeable, extravert, conscientious, open to experiences, neurotic). While self-reported physical activity is typically considered a limitation over objective measurement (for example, accelerometers) [23], with the IPAQ lacking evidence of validity and reliability for people with disabilities and for people who use mobility devices, research suggests that more work needs to be done on improving interpretation of accelerometer output, at least among those with possible mobility disabilities [24]. This is further complicated by variation in the types and severity of disabilities [25]. Despite this, some self-report physical activity instruments have been developed for people with disabilities [13].

The HILDA Survey restricted sampling to residents of private dwellings, thereby excluding residents of institutions, notably hospitals and other health care institutions [10]. People with severe disabilities are also less likely to participate in HILDA. This could bias results if people with a severe disability are less likely to engage in physical activity and tend to live near each other. Due to the broad definition of disability (such as the lack of information on disability type), the group of people who report having a disability in the HILDA Survey is likely to be diverse, covering a wide range of activity limitations and impairments. Future research is required on whether there is substantive variation in physical activity by type of disability, as this could guide potential policy options.

In the context of social-ecological models, factors relating to physical activity participation among people living with disabilities have been discussed [2]. These factors exist at the intrapersonal, interpersonal, institutional, community and policy levels [2]. As the type and severity of disability can be diverse (intellectual disability vs physical disability), factors independent of geographical location (at the intrapersonal level- attitudes, benefits and perceived benefits of physical activity) are likely to have greater influence on physical activity levels [2]. This must be taken into account when interpreting the results of this study. Expanding the current study to examine specific impairments (intellectual, mental, physical, or sensory or their combinations) may be more useful and allow for more targeted interventions to increase physical activity levels for people living with disability.

There are several priorities for future research. Australian State and Territory governments establish frameworks for urban design, which are interpreted and implemented by LGAs. Reviewing differences in state-level urban design policies related to disability and physical activity, and their implementation at the LGA-level, would provide a greater understanding of policies that might influence physical activity among people with a disability, and whether these equated to on-the-ground environmental differences.

## Conclusions

We did not find evidence that the association between disability and physical activity varied across local government areas in Australia. These findings suggest the need for ‘whole-of-government’ approaches to reducing inequalities in physical activity, rather than solely those at the local government level. This study could be of interest due to the growing interest in health inequalities, and how local government policy might, or might not, contribute to these inequalities.

## Data Availability

HILDA data is available on request from the Australian Data Archive.
